# The noncoding and coding transcriptional landscape of the peripheral immune response in patients with COVID‐19

**DOI:** 10.1002/ctm2.200

**Published:** 2020-10-11

**Authors:** Hao Tang, Yuehan Gao, Zhaohuai Li, Yushan Miao, Zhaohao Huang, Xiuxing Liu, Lihui Xie, He Li, Wen Wen, Yingfeng Zheng, Wenru Su

**Affiliations:** ^1^ Department of Respiratory and Critical Care Medicine Changzheng Hospital Second Military Medical University Shanghai China; ^2^ Department of Critical Care Wuhan Huo Shen Shan Hospital Hubei China; ^3^ State Key Laboratory of Ophthalmology, Zhongshan Ophthalmic Center Sun Yat‐sen University Guangzhou China; ^4^ National Center for Liver Cancer Second Military Medical University Shanghai China

**Keywords:** blood, COVID‐19, microRNAs, noncoding RNAs

## Abstract

**Background:**

COVID‐19 is currently a global pandemic, but the response of human immune system to severe acute respiratory syndrome coronavirus 2 (SARS‐CoV‐2) infection remains unclear. Noncoding RNAs serve as immune regulators and thus may play a critical role in disease progression.

**Methods:**

We performed multi‐transcriptome sequencing of both noncoding RNAs and mRNAs isolated from the red blood cell depleted whole blood of moderate and severe COVID‐19 patients. The functions of noncoding RNAs were validated by analyses of the expression of downstream mRNAs. We further utilized the single‐cell RNA‐seq data of COVID‐19 patients from Wilk et al. and Chua et al. to characterize noncoding RNA functions in different cell types.

**Results:**

We defined four types of microRNAs with different expression tendencies that could serve as biomarkers for COVID‐19 progress. We also identified miR‐146a‐5p, miR‐21‐5p, miR‐142‐3p, and miR‐15b‐5p as potential contributors to the disease pathogenesis, possibly serving as biomarkers of severe COVID‐19 and as candidate therapeutic targets. In addition, the transcriptome profiles consistently suggested hyperactivation of the immune response, loss of T‐cell function, and immune dysregulation in severe patients.

**Conclusions:**

Collectively, these findings provide a comprehensive view of the noncoding and coding transcriptional landscape of peripheral immune cells during COVID‐19, furthering our understanding and offering novel insights into COVID‐19 pathogenesis.

## Graphical abstract

Highlight

1. miR‐146a‐5p, miR‐21‐5p, and miR‐142‐3p are potential biomarkers of, and therapeutic targets for COVID‐19.

2. Several miRNAs, such as miR‐15b‐5p, are specific for severe COVID‐19 and may serve as potential biomarkers and therapeutic targets.

3. The blood transcriptome profiles suggest hyperactivation of the immune response, loss of T‐cell function, and immune dysregulation in patients with severe COVID‐19.

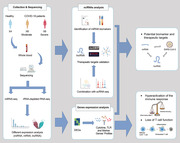

**Abbreviations**
ACE2: angiotensin I converting enzyme 2
AIM2: absent in melanoma 2
ARDS: acute respiratory distress syndrome
BALF: bronchoalveolar lavage fluid
CCL20: C‐C motif chemokine ligand 20
CLIP: cross‐linking and immunoprecipitation
COVID‐19: coronavirus disease 2019
DEGs: differentially expressed genes
DElncRs: differentially expressed lncRNAs
DEmiRs: differentially expressed miRNAs
IFN: interferon
IL: interleukin
IL6ST: interleukin 6 signal transducer
IRAK: interleukin‐1 receptor‐associated kinase
lncRNAs: long ncRNAs
MALAT1: metastasis associated lung adenocarcinoma transcript 1
MTI: miRNA‐target interaction
MTMR3: myotubularin‐related protein 3
ncRNAs: noncoding RNAs
NEAT1: nuclear paraspeckle assembly transcript 1
NF‐κB: nuclear factor‐κB
PBMCs: peripheral blood mononuclear cells
SARS‐CoV‐2: severe acute respiratory syndrome coronavirus 2
scRNA‐seq: single‐cell RNA sequencing
TLR: Toll‐like receptor
TRAF6: tumor necrosis factor receptor‐ associated factor 6

## 1 BACKGROUND

The coronavirus disease 2019 (COVID‐19) outbreak caused by severe acute respiratory syndrome coronavirus 2 (SARS‐CoV‐2) has soon become a global pandemic resulting in numerous deaths, and has been declared as a public health emergency of international concern.^1^ Global researchers are currently making great efforts to understand the pathogenesis of COVID‐19. By characterization of immune cell transcriptomes, several recent studies have demonstrated that heightened immune responses across innate and adaptive immune system could contributed a lot to disease severity.^2^ The abnormal host responses could be associated with aberrant immune cell activation including T cells, monocytes, and macrophages, as well as dysregulated cytokine production.^3^, ^4^ Despite the significant efforts by researchers and clinicians, there are still no effective clinical treatments or specific vaccines for COVID‐19.^5^, ^6^, ^7^

Noncoding RNAs (ncRNAs) are a class of RNAs not involved in protein production and can be subdivided into small (miRNAs, tRNAs, PIWI‐targeting RNAs) and long ncRNAs (lncRNAs), based upon their size.^8^ This class of RNAs not only regulates fundamental biological processes including immune system development and regulation, but also plays a critical role in multiple human diseases. Accumulating evidence has demonstrated that miRNAs could influence the replication and pathogenesis of RNA viruses through direct binding to the viral genome (miR‐122 interacts with the hepatitis C virus genome and inhibits viral RNA degradation^9^) or by inducing changes in the host transcriptome (increased miR‐146a expression during dengue virus infection negatively regulated the host response^10^).^11^ Additionally, miRNAs have been recognized as novel disease markers owing to their tissue specificity, stability, and association with clinicopathological parameters.^12^, ^13^ lncRNAs may act as upstream regulators of miRNAs, serving as “sponges” that compete for miRNA binding and reverse the regulatory effect of miRNAs on target mRNAs.^8^ Therefore, altered levels of ncRNAs during progression of COVID‐19 could constitute a critical component of the host response, reflecting distinct phases of the antiviral immune response from disease onset to recovery. Given their critical roles in disease pathogenesis, ncRNAs could serve as biomarkers, and even as novel therapeutic targets for COVID‐19.

However, little is known about the ncRNA transcriptome of red blood cell (RBC) depleted whole blood in COVID‐19 patients and its potential clinical value. A comprehensive analysis of COVID‐19 ncRNA profile is urgently needed to gain a deeper understanding of disease pathogenesis and to discover more effective strategies for diagnosis and treatment. Here, we collected RBC‐depleted whole blood samples from patients with severe and moderate COVID‐19, and performed multi‐transcriptome sequencing of ncRNAs and mRNAs.

## 2 METHODS

### 2.1 Study approval

The study was approved by the Ethics Committee of the Huo Shen Shan Hospital of Wuhan. All blood samples for multi‐transcriptome sequencing were existing samples that were collected during standard COVID‐19 treatment process, with no extra burden posed.

### 2.2 Sample collection

Whole blood was obtained from six severe and six moderate COVID‐19 patients at Huo Shen Shan Hospital of Wuhan during standard diagnostic tests. Sample collection criteria included age ≥18 years and admission to Huo Shen Shan Hospital (wards and ICU) with a positive result in SARS‐CoV‐2 nasopharyngeal swab RT‐PCR test. For controls, blood was collected from four uninfected adult donors with a negative nasopharyngeal swab. The classification of COVID‐19 severity was based on WHO guidelines. The classification of acute respiratory distress syndrome (ARDS) was based on the Berlin criteria (acute onset of hypoxemic respiratory failure with a PaO_2_/FiO_2_ < 300 on at least 5 cm of positive end‐expiratory pressure, bilateral infiltrates on chest X‐ray). Ages were shown as ranges to protect the privacy of patients and healthy controls. All donors were asked for consent for genetic research.

### 2.3 mRNA and miRNA isolation and purification

Erythrocytes were removed from human whole blood with Erythrocyte lysis buffer (Cat. No.: AR1118, BOSTER). Small RNAs (<200 nt) and large RNAs (>200 nt) were isolated using the NucleoSpin miRNA kit (Macherey‐Nagel, Düren, Germany) following the manufacturer's instructions. RNA purity was checked using the NanoPhotometer spectrophotometer (IMPLEN, CA). RNA concentration was measured using Qubit RNA Assay Kit in Qubit 2.0 Flurometer (Life Technologies, CA). RNA integrity was assessed using the RNA Nano 6000 Assay Kit of the Bioanalyzer 2100 system (Agilent Technologies, CA).

### 2.4 Library construction and sequencing

For rRNA‐depleted RNA‐seq sample preparations, we used 3 μg RNA per sample. Sequencing libraries were generated using the rRNA‐depleted RNA by NEBNext Ultra Directional RNA Library Prep Kit for Illumina (NEB) following manufacturer's instructions. The libraries were sequenced on an Illumina Hiseq 4000 platform and 150 bp paired‐end reads were generated.

For miRNA‐seq, we used 1 μg RNA per sample for small RNA library construction. Sequencing libraries were generated using NEBNext Multiplex Small RNA Library Prep Set for Illumina (NEB) following manufacturer's instructions. To acquire sufficient sequencing coverage, four samples were combined into one lane and two technical replicates were ran for each library using multiplexing. T cluster generation was performed on a Flow Cell v3 (TruSeq SR Cluster Kit v3; Illumina) using cBOT. The library preparations were sequenced on an Illumina Hiseq 2500 platform and 50 bp single‐end reads were generated.

### 2.5 Data processing

Quality control processes consisted of adapter trimming, low‐quality reads removal with cutadapt software (version 2.10; https://cutadapt.readthedocs.io/en/stable/), and rRNA and tRNA removal. The total rRNA proportion indicated the quality of our samples with all samples (small and large RNA) containing rRNA less than 5%. All clean large RNA data were mapped to the human genome GRCh38 using HISAT2 (version 2.2.0; http://daehwankimlab.github.io/hisat2/). miRNA data were mapped to the miRBase (version 22; http://www.mirbase.org/) with Bowtie software (version 1.2.3; www.sourceforge.net/projects/bowtie-bio/files/bowtie), allowing 0 mismatch and mapping with the proximity of mature miRNAs. Bam files were sorted by Samtools (version 1.9; http://samtools.sourceforge.net/index.shtml). Gene counts were generated using the featureCounts program, part of the Subread package (version 2.0.0; http://subread.sourceforge.net/). miRNA counts were summarized with perl scripts written by authors of this paper.

The quality control of raw read data was done by FastQC (version 0.11.9) and multiQC (version v1.8). For rRNA‐depleted RNA‐seq data, the average number of reads per sample is about and the quality score for each sample is between 33 and 37, indicating good raw data quality. For miRNA‐seq data, two peaks at 22 nt and 33 nt were observed in read length distribution, with the first peak greater than the second peak. All RNA sequencing data exhibited rRNA alignment rates smaller than 5%. Sequencing data are available in the National Genomic Data Center (NGDC) (primary accession number HRA000238).

### 2.6 miRNA‐mRNA and lncRNA‐miRNA network construction

Both miRNA‐mRNA and lncRNA‐miRNA networks were constructed with Cytoscape (version 3.7.2; https://cytoscape.org/) based on their interaction. The R package “multimiR” (version 2.3; http://multimir.org) were used to identify miRNA‐mRNA interaction. We filtered every miRNA‐mRNA pair using the most stringent criteria, including validation by luciferase experiments and functional miRNA‐target interaction (MTI) tests. Database ENCORI (version; http://starbase.sysu.edu.cn/) were used to identify lncRNA‐miRNA interaction validated by cross‐linking and immunoprecipitation (CLIP)‐sEquation (≥5).

### 2.7 Single‐cell RNA‐seq computational pipelines and analysis

The R package Seurat (version 3.0; https://satijalab.org/seurat/) was used for single‐cell analysis including normalization, scaling, dimensionality reduction, clustering, transcriptome analysis, and visualization. Peripheral blood mononuclear cells (PBMCs) single‐cell RNA sequencing (scRNA‐seq) data (count matrix) from Wilk et al and nasopharynx scRNA‐seq data (Seurat object) from Chua et al were obtained from the provided available data sources for co‐analysis. PBMC data scaling, integration, clustering, and dimensionality reduction were performed following the R scripts provided by Wilk et al. Seurat's implementation of the Wilcoxon rank‐sum test (FindMarkers()) was used to determine differentially expressed genes (DEGs) for each cluster of both datasets. Cellular identity was determined based on marker gene expression presented in Supporting Information figures during re‐clustering.

### 2.8 Statistic

The power calculation was performed with the RNAseqPS tool^14^ (https://cqs-vumc.shinyapps.io/rnaseqsamplesizeweb/). The number of patients offer sufficient power to detect twofold changes in gene expression based on depth and coverage of our sequencing data. The total quantity of each RNA was used to calculate the number of mapped reads per kilobase per million reads (RPKM). differentially expressed miRNAs (DEmiRs), DEGs, and differentially expressed lncRNAs (DElncRs) were reported using an adjusted *P* value threshold of .05, and a minimum fold change (FC) of 2. The adjusted *P* values were obtained from Independent Hypothesis Weighting as implemented in the DESeq2 package. The *P*‐values shown in each box plot figures are exact two‐sided by one‐way ANOVA with post hoc comparisons by Tukey's test using GraphPad Prism 8. Exact two‐sided *P*‐values and the 95%CI by Pearson correlation coefficients for each correlation are shown in scatter plots by GraphPad Prism 8.

## 3 RESULTS

### 3.1 Sample collection and transcriptome sequencing

**FIGURE 1:**
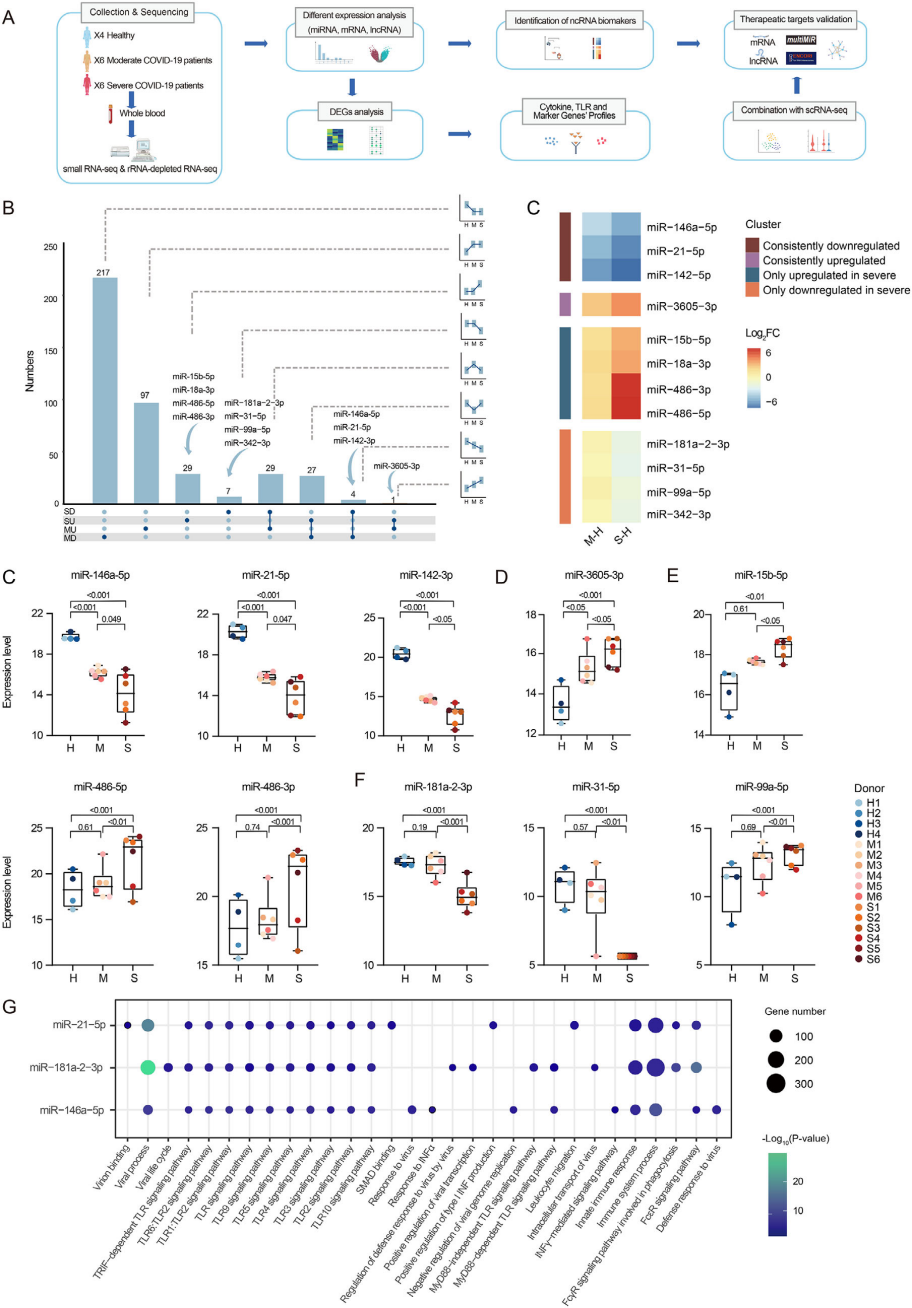
**Identification of biomarker miRNAs by small RNA‐sEquation. (A)** Research experimental design. We collected red blood cell‐depleted whole blood from moderate, severe COVID‐19 patients and healthy donors. Small RNA‐seq and rRNA depleted‐seq were performed. Biomarker and therapeutic target miRNAs were identified and validated with co‐analysis of mRNAs and lncRNAs and combination of scRNA‐seq data. Transcriptome during viral infection was studied. **(B)** UpSet plot shows the number of DEmiRs with eight different expression tendencies. The box and line charts show the tendency of each group. The arrows point out the position of biomarker miRNAs. **(C)** The heatmap shows the expression levels of the biomarker miRNAs in four specific DEmiR expression tendency. **(D‐G)** Expression of biomarker miRNAs in each sample. Each plot is colored by donor of origin. The X axes accord with the COVID‐19 status of each donor: M (n = 6), S (n = 6), and H (n = 4). The *P*‐values are exact two‐sided generated by one‐way ANOVA with post hoc comparisons by Tukey's test. Boxplot features: minimum whisker, the smallest value within; minimum box, 25th percentile; center, median; maximum box, 75th percentile; maximum whisker, the largest value within. **(D)** miRNAs consistently downregulated. **(E)** miRNAs consistently upregulated. **(F)** miRNAs only upregulated in severe COVID‐19 patients. **(G)** miRNAs only downregulated in severe patients. **(H)** GO‐term functional enrichment by biological progress for the predicted target genes of three representative miRNAs

RBC‐depleted whole blood samples were obtained from laboratory‐confirmed moderate (n = 6) and severe COVID‐19 patients (n = 6), as well as healthy controls (n = 4) (Figure 1A). Table 1 shows the detailed clinical characteristics of patients. Notably, the three groups exhibited no statistically significant difference in age between each other (Figure S2B).

**TABLE 1:** Sample characteristics and clinical features of patients with COVID‐19

ID	Gender	Age (years)	Disease	Virus (sequencing)	Clinical/laboratory results	PaO_2_/FIO_2_	ARDS	ICU
M1	Male	50‐59	COVID‐19	SARS‐CoV‐2	SARS‐CoV‐2	–	N	N
M2	Female	70‐79	COVID‐19	SARS‐CoV‐2	SARS‐CoV‐2	–	N	N
M3	Male	50‐59	COVID‐19	SARS‐CoV‐2	SARS‐CoV‐2	–	N	N
M4	Female	80‐89	COVID‐19	SARS‐CoV‐2	SARS‐CoV‐2	–	N	N
M5	Male	20‐29	COVID‐19	SARS‐CoV‐2	SARS‐CoV‐2	–	N	N
M6	Male	70‐79	COVID‐19	SARS‐CoV‐2	SARS‐CoV‐2	–	N	N
S1	Female	70‐79	COVID‐19	SARS‐CoV‐2	SARS‐CoV‐2	99	Y	Y
S2	Male	70‐79	COVID‐19	SARS‐CoV‐2	SARS‐CoV‐2	160	Y	Y
S3	Male	60‐69	COVID‐19	SARS‐CoV‐2	SARS‐CoV‐2	124	Y	Y
S4	Male	80‐89	COVID‐19	SARS‐CoV‐2	SARS‐CoV‐2	134	Y	N
S5	Male	60‐69	COVID‐19	SARS‐CoV‐2	SARS‐CoV‐2	84	Y	Y
S6	Male	70‐79	COVID‐19	SARS‐CoV‐2	SARS‐CoV‐2	131	Y	Y
H1	Female	50‐59	Healthy	NA	Negative	–	NA	NA
H2	Male	60‐69	Healthy	NA	Negative	–	NA	NA
H3	Female	50‐59	Healthy	NA	Negative	–	NA	NA
H4	Male	60‐69	Healthy	NA	Negative	–	NA	NA

We applied small RNA‐seq and rRNA‐depleted RNA‐seq on each blood sample. After quality control, reads were mapped to miRbase and GRCh38 genome, respectively. The count matrixes derived from the latter were further separated into protein‐coding mRNAs and lncRNAs.

We identified differentially expressed miRNAs (DEmiRs), DEGs, and DElncRs from comparisons between moderate‐healthy (M‐H), severe‐healthy (S‐H), and severe‐moderate (S‐M) (Figure S1). The list of DEmiRs, DEGs, and DElncRs was determined using adjusted *P* values (*q*‐value < 0.05) and FC ratios (|log2FC| ≥ 1) (Table S1‐S3).

Considering the total number of identified mRNAs and ncRNAs, miRNAs had the most evident alterations, supporting their high sensitivity as potential biomarkers (Figure S2A).

### 3.2 Identification of biomarker miRNAs by small RNA‐seq

The DEmiRs of each group are depicted in the heatmap (Figure S2B) and subdivided into different types based on their expression tendencies (Figure 1B). Four types of miRNAs were defined as candidate miRNA biomarkers (Figure 1C): (a) miRNAs consistently downregulated, including miR‐146a‐5p, miR‐21‐5p, and miR‐142‐3p (Figure 1D); (b) miRNAs consistently upregulated, including miR‐3605‐3p (Figure 1E); (c) miRNAs upregulated only in patients with severe COVID‐19 with no statistically significant difference in M‐H comparison, including miR‐15b‐5p, miR‐486‐3p, and miR‐486‐5p (Figure 1F); and (d) miRNAs downregulated only in severe cases, including miR‐181a‐2‐3p, miR‐31‐5p, and miR‐99a‐5p (Figure 1G).

Functional enrichment analysis of the predicted target genes for several representative biomarker miRNAs showed high correlation with inflammation and antiviral immune responses (Figure 1H). Processes including “virus binding,” “virus process,” and “defense response to virus” implied miRNA engagement in viral infection. These miRNAs were also associated with several Toll‐like receptor (TLR) signaling pathways, as well as production of and response to interferon.

### 3.3 Bulk RNA‐seq suggests biomarker miRNAs as potential therapeutic targets

**FIGURE 2:**
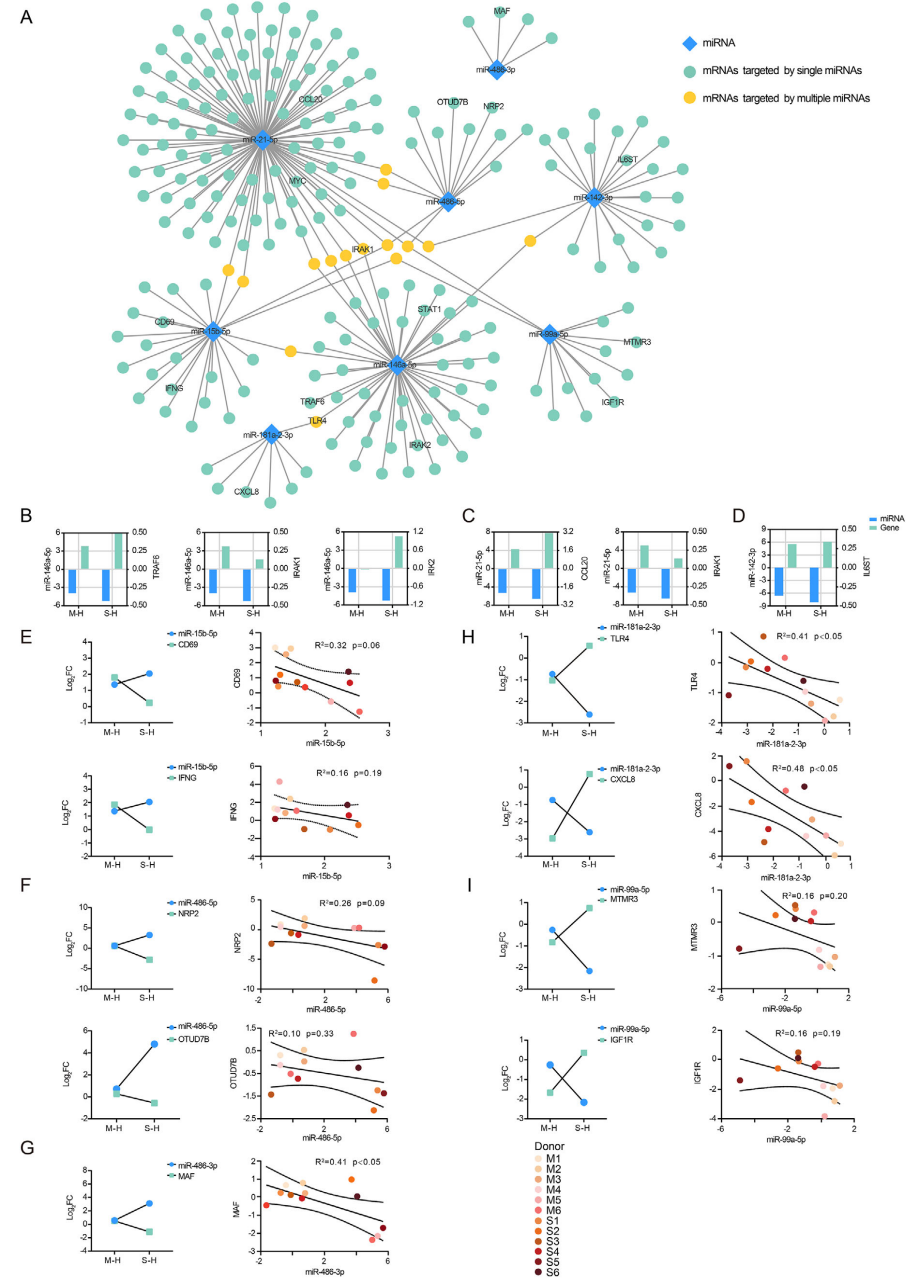
**Validation of therapeutic target miRNAs by mRNAs. (A)** An integrated miRNA‐mRNA regulatory network. Only pairs mentioned in the text were labeled. **(B‐D)** Expression of consistently downregulated miRNAs and target mRNAs in moderate and severe groups compared to healthy controls. The left and right Y axes correspond to the log_2_FC of miRNAs and mRNAs, respectively. **(B)** Expression of miR‐146a‐5p and downstream *TRAF6*, *IRAK1* and *IRAK2*. **(C)** Expression of miR‐21‐5p and downstream *CCL20* and *IRAK1*. **(D)** Expression of miR‐142‐3p and downstream *IL6ST*. **(E‐I)** Expression and correlation of severe COVID‐19‐specific miRNAs and target mRNAs. The results were shown as log_2_FC comparing S, M groups to healthy controls. Scatter plots show exact two‐sided *P*‐values and the 95% confidence interval (CI) by Pearson correlation coefficients for each correlation. The number of samples: M (n = 6) and S (n = 6). Each plot is colored by sample of origin. **(E)** Expression and correlation of miR‐15b‐5p and downstream *CD69* and *IFNG*. **(F)** Expression and correlation of miR‐486‐5p and downstream *NRP2* and *OTUD7B*. **(G)** Expression and correlation of miR‐486‐3p and *MAF*. **(H)** Expression and correlation of miR‐181a‐2‐3p and downstream *TLR4* and *CXCL8*. **(I)** Expression and correlation of miR‐99a‐5p and downstream *MTMR3* and *IGF1R*

To better understand the function of biomarker miRNAs, we retrieved their validated downstream mRNAs, analyzed the miRNA‐mRNA correlation, and constructed an integrated miRNA‐mRNA regulatory network with “multimiR” package (Figure 2A). Of note, we filtered every miRNA‐mRNA pair with the most stringent criteria, including validation by luciferase experiments and functional MTI tests.

Consistent downregulation of miR‐146a‐5p, miR‐21‐5p, and miR‐142‐3p promotes inflammatoty process.^15^, ^16^, ^17^ The miR‐146a‐5p negatively correlated with downstream target mRNAs interleukin‐1 receptor‐associated kinase 1 (*IRAK1*), *IRAK2*, and tumor necrosis factor receptor‐ associated factor 6 (*TRAF6*), which participate in the nuclear factor‐κB (NF‐κB) pro‐inflammatory signaling pathway^18^, ^19^, ^20^ (Figure 2B). miR‐21‐5p may directly target *IRAK1* and chemokine C‐C motif chemokine ligand 20 (*CCL20*), which was upregulated in inflamed airway epithelium^21^ (Figure 2C). Decreased miR‐142‐3p induces production of glycoprotein 130 (gp130), an activator of JAK/STAT signaling pathway, by binding to interleukin 6 signal transducer (*IL6ST*) mRNA^22^ (Figure 2D).

miRNAs that were up‐ or downregulated only in severe cases may contribute to COVID‐19 deterioration. Upregulated miR‐15b‐5p seemed to play dual roles. First, it negatively correlated with *IFNG* and *CD69* that were involved in T‐cell function and activation^23^, ^24^ (Figure 2E). Moreover, miRNAs can promote RNA virus replication by binding to and stabilizing the viral genome. A recent research identified miR‐15b‐5p as the most likely candidate to target SARS‐CoV‐2 genome with the highest target score and binding sites^25^ (Figure S2C). Upregulation of miR‐15b‐5p could accelerate intracellular viral replication, promote cell‐to‐cell dissemination, mediate virus‐induced transcriptome changes, and ultimately intensify the severity of COVID‐19.

Other upregulated candidate miRNAs also contributed to COVID‐19 pathogenesis. miR‐486‐5p not only targets neuropilin 2 (*NRP2*) encoding inflammatory inhibitor neuropilin‐2, but also represses *OTUD7B* to induce excessive inflammation in lung^26^, ^27^, ^28^ (Figure 2F). Another miRNA miR‐486‐3p directly targets *MAF*, and downregulation of *MAF* may result in immune response dysregulation^29^, ^30^ (Figure 2G).

Among the miRNAs downregulated in severe cases was miR‐181a‐2‐3p, a serum biomarker of chronic obstructive pulmonary disease.^31^ Downregulation of miR‐181a‐2‐3p is also associated with enhanced *TLR4* and *CXCL8* expression^32^, ^33^ (Figure 2H). Another downregulated miRNA was miR‐99a‐5p targeting the proinflammatory genes, insulin like growth factor 1 receptor (*IGF1R*)^34^, ^35^ and myotubularin‐related protein 3 (*MTMR3*), which were reported to induce weaker antiviral immunity^36^, ^37^ (Figure 2I).

Overall, the results of our detailed functional miRNA‐mRNA analysis suggest that the candidate biomarker miRNAs identified may contribute to COVID‐19 pathogenesis and serve as therapeutic targets.

### 3.4 Validation of miRNAs as therapeutic targets by single cell RNA‐seq

**FIGURE 3:**
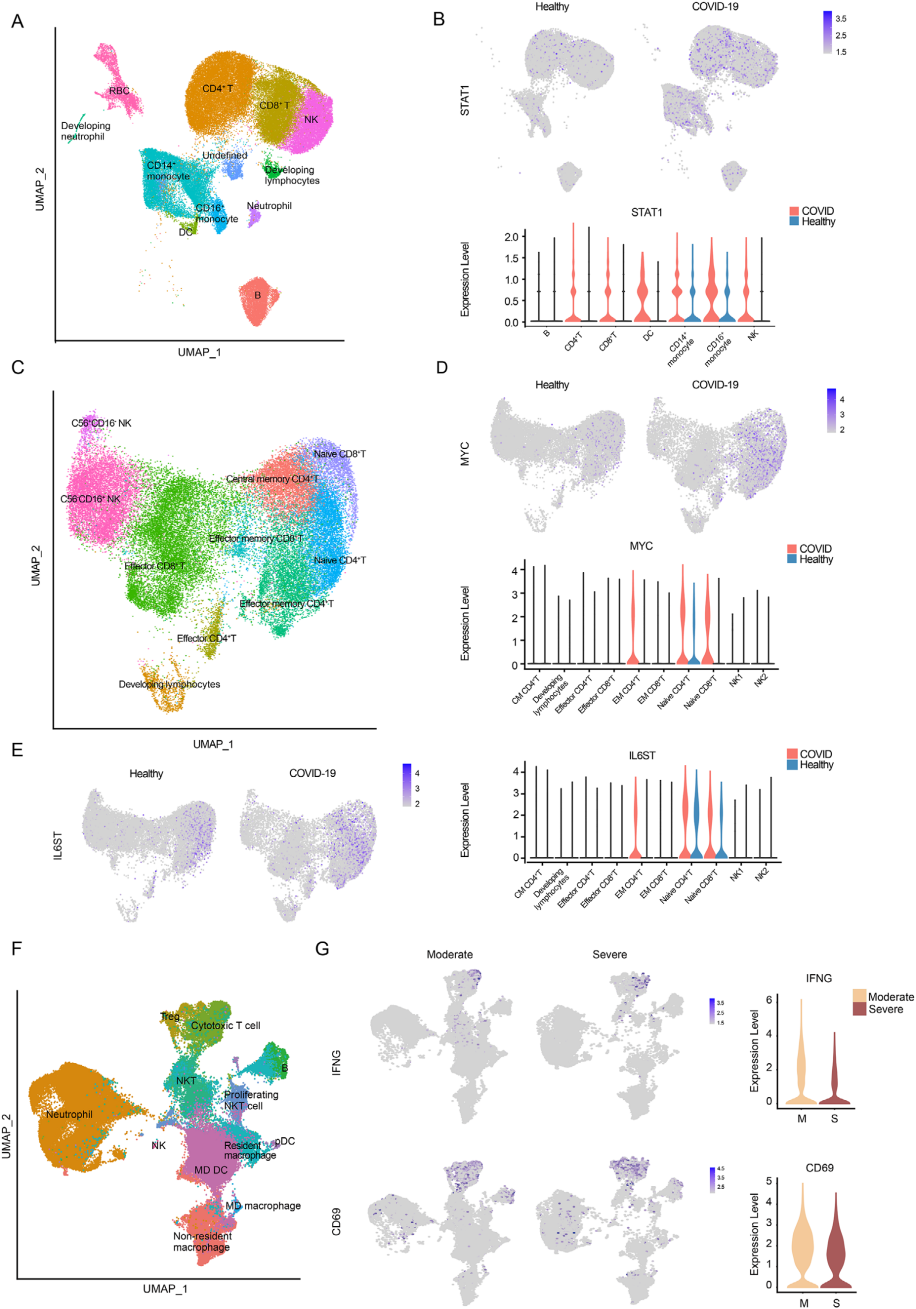
**Validation of therapeutic target miRNAs by scRNA‐sEquation. (A)** UMAP plot of dataset by Wilk et al colored by clusters labeled with cell type annotation. **(B)** UMAP embedding of PBMCs and violin plot shows *STAT1* expression in patients and healthy controls. **(C)** UMAP plot of T cells and NK cells colored by clusters labeled with cell type annotation. **(D)** UAMP and violin plot depicts *MYC* expression in different types of cells in patients and healthy controls. **(E)** UAMP and violin plot depicts *IL6ST* expression in different types of cells in patients and healthy controls comparison. **(F)** UMAP embedding of immune cells transported from peripheral blood colored by clusters labeled with manual cell type annotation. **(G)** UAMP and violin plots show expression of *IFNG* and *CD69* in the nasopharyngeal cells in severe and moderate patients

To further validate the function of candidate miRNAs, we used the scRNA‐seq data from Wilk et al, pertaining to the sequencing of PBMCs from severe cases and healthy controls.^3^ The samples from Wilk et al study could result in different DEGs with ours because of the absence of neutrophils. We successfully identified 13 major peripheral blood cell clusters (Figure 3A). Monocytes appeared to be the most remodeled cells (Figure S3A). The major groups of immune cells were re‐clustered for further analysis (Figure S3B).

We first focused on consistently downregulated miRNAs. Our analysis revealed broad upregulation of *STAT1* targeted by miR‐146a‐5p that encodes a key element of the JAK/STAT pathway^38^, ^39^ (Figure 3B). As for miR‐21‐5p, *MYC*, a T‐cell activation marker, displayed upregulation specifically in naïve T cells^40^, ^41^ (Figure 3C and D). *IL6ST*, a target of miR‐142‐3p, was upregulated mainly in naïve T cells, suggesting increased sensitivity to interleukin (IL)‐6 signaling^42^ (Figure 3E). Upregulation of *STAT1*, *MYC*, and *IL6ST* was also observed in our bulk RNA‐seq data with smaller fold changes and larger adjusted *P* value (Figure S3C).

It was improper to use the scRNA‐seq data by Wilk et al. that only addressed the S‐H comparison to validate the function of miRNAs differentially expressed in the S‐M comparison. Neutrophils were also excluded from PBMC samples. Thus, we used the scRNA‐seq data of Chua et al obtained in cells of the nasopharyngeal area of severe and moderate COVID‐19 patients, and healthy controls.^4^ We focused on cells transported from the peripheral blood to the nasopharyngeal area, whose transcriptome tended to be regulated by blood biomarker miRNAs (Figure 3F). The targets of miR‐15b‐5p, *IFNG*, and *CD69* were downregulated in nasopharyngeal CD8+ T cells in S‐M comparison. We also observed the upregulation of miR‐99a‐5p target, MTMR3 as well as miR‐181a‐2‐3p targets, *TLR4* and *CXCL8*, in neutrophils in the context of severe disease (Figure S3D and E).

Re‐examining miR‐146a‐5p, miR‐21‐5p, and miR‐142‐3p targets with Chua et al data successfully identified upregulation of *IRAK1* and *IRAK2* along with other targets mainly expressed in neutrophils^43^ (Figure S4).

Overall, the combined analysis of our miRNA data and two published scRNA‐seq datasets further validated these candidate biomarker miRNAs as potential therapeutic targets in specific cell types.

### 3.5 lncRNAs could be the upstream “sponges” inhibiting miRNA function

lncRNAs may act as miRNA sponges that bind to specific miRNA sites, reduce miRNA‐mRNA interaction, and inhibit the regulatory function of miRNAs.^8^ To provide a comprehensive overview of the upstream regulator of our potential therapeutic target miRNAs, we established a lncRNA‐miRNA network based on lncRNA‐miRNA CLIP results.

**FIGURE 4:**
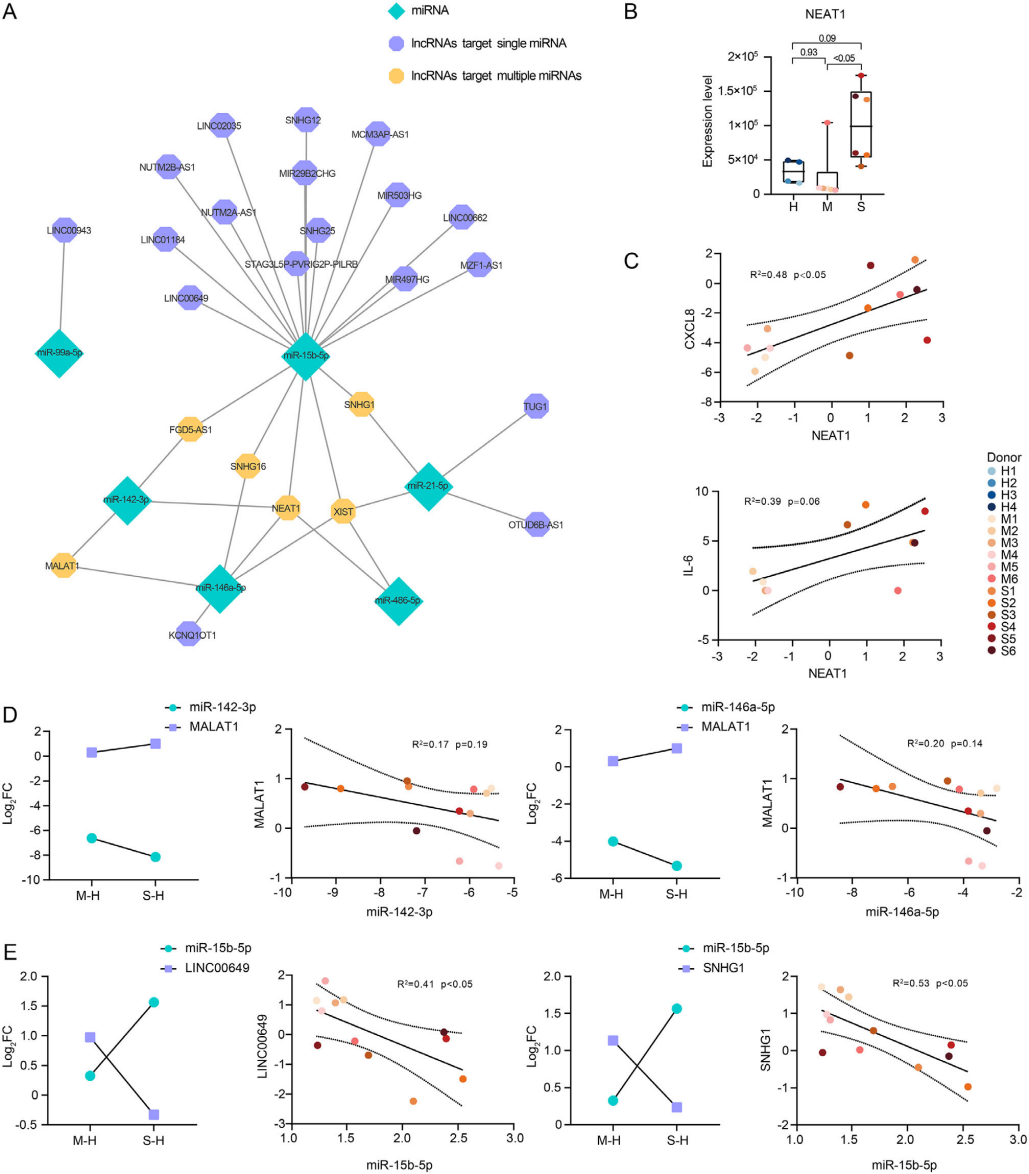
**Validation of therapeutic target miRNAs by lncRNA. (A)** A lncRNA‐miRNA regulatory network. **(B)** Expression of *NEAT1* in each sample. Each plot is colored by sample of origin. The *P*‐values are exact two‐sided generated by one‐way ANOVA with post hoc comparisons by Tukey's test. Boxplot features: minimum whisker, the smallest value within; minimum box, 25th percentile; center, median; maximum box, 75th percentile; maximum whisker, the largest value within. The number of samples: M (n = 6) and S (n = 6). **(C)** Expression and correlation of upstream *NEAT1* and downstream *CXCL8* and IL‐6. The results were shown as log_2_FC comparing S, M groups to H group. The number of samples for *NEAT1* and *CXCL8*: M (n = 6) and S (n = 6). The number of samples for *NEAT1* and IL‐6: M (n = 5) and S (n = 5). **(D‐E)** Expression and correlation of upstream lncRNAs and downstream miRNAs. The results were shown as log_2_FC comparing S, M groups to H group. Scatter plots show exact two‐sided *P*‐values and the 95% CI by Pearson correlation coefficients for each correlation. The number of samples: M (n = 6) and S (n = 6). Each plot is colored by donor of origin. **(D)** The correlation between lncRNA *MALAT1* and downstream miR‐142‐3p and miR‐146a‐5p. **(E)** The correlation between lncRNA *LINC00649* and miR‐15b‐5p, and lncRNA *SNHG1* and miR‐15b‐5p

DElncRs in each group were shown in the heatmap (Figure S5A). We explored lncRNA‐miRNA interactions using the StarBase database, set up filter criteria (CLIP ≥ 5), and applied it to our lncRNA data (Figure 4A). Nuclear paraspeckle assembly transcript 1 (*NEAT1*) seemed to dominate the networks with four associated miRNAs, implying its critical role in COVID‐19 pathogenesis. Correspondingly, *NEAT1* showed increased expression across S‐M and S‐H comparisons, with no significant change in the M‐H comparison (Figure 4B). Moreover, *NEAT1* might increase the production of inflammatory cytokines, IL‐6 and CXCL8, which confirmed its critical role in inflammation^44^, ^45^ (Figure 4C).

We identified several lncRNA‐miRNA pairs with opposite expression tendencies and biological functions. Both miR‐146a‐5p and miR‐142‐3p were negatively correlated with a canonical inflammatory inhibitor, metastasis associated lung adenocarcinoma transcript 1 (*MALAT1*)^46^ (Figure 4D). *MALAT1* could absorb miR‐146a‐5p and miR‐142‐3p to repress their anti‐inflammatory function.^47^, ^48^ miR‐15b‐5p could be targeted by multiple lncRNAs, among which inflammatory inhibitors, long intergenic nonprotein coding RNA 649 (*LINC00649*) and small nucleolar RNA host gene 1 (*SNHG1*), showed opposite expression patterns^49^ (Figure 4E).

Targeting these lncRNAs could affect the silencing function of their downstream miRNAs, which provides another treatment strategy for COVID‐19.

### 3.6 Cytokines, TLR, and marker gene profiles

**FIGURE 5:**
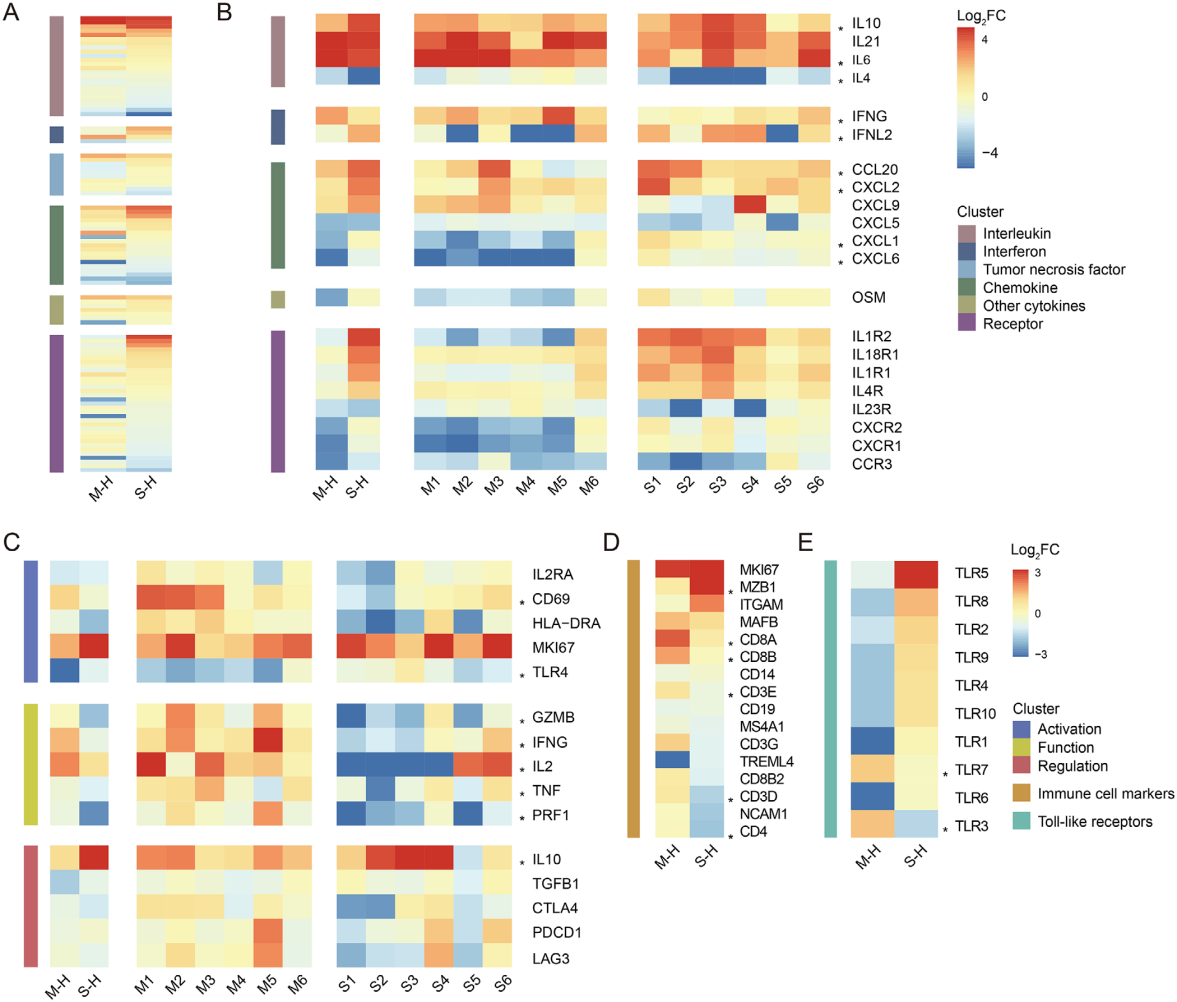
**Cytokine, TLR, and marker genes profiles. (A)** Heatmap of cytokine‐related genes expression. **(B)** Heatmap of selected cytokine‐related genes. Relative expression of each patients compared to healthy controls was shown on the right. **(C)** Heatmap of genes participated in the activation, function, and regulation process of T cells. Relative expression of each patients compared to healthy controls was shown on the right. **(D)** Heatmap of immune cell marker genes. **(E)** Heatmap of *TLR* family

Elevated inflammatory mediators play a crucial role in fatal pneumonia caused by SARS‐CoV‐2.^2^ To better understand the potential effect of each type of cytokines, we sorted genes into six categories including “interleukin,” “interferon,” “chemokine,” “tumor necrosis factor,” “other,” and “receptor” of which “interleukin” and “chemokine” displayed the most evident changes in gene expression (Figure 5A).

ILs exhibited the largest fold changes (Figure 5B). Consistent with previous large‐scale clinical studies, we observed significant upregulation of *IL10* and *IL6* in COVID‐19 patients, suggesting the ongoing hyperactivation of inflammatory effects.^50^, ^51^, ^52^ In addition, we observed a consistent downregulation of *IL4* that limits tissue damage during the immune response.^53^

We detected consistent upregulation of *CXCL2* critical for recruiting neutrophils into inflamed lungs.^54^ The expression of another important proinflammatory cytokine *CCL20* was upregulated. We also observed increased expression of *CXCL1* and *CXCL6* in S‐M comparison, indicating an excessive activation of inflammatory effects in severe cases^55^, ^56^ (Figure S6).

The downregulation of *IFNG* and *CD69* in severe cases (probably regulated by miR‐15b‐5p) led us to T‐cell exhaustion analyses. This downregulation was also observed in PBMCs, bronchoalveolar lavage fluid (BALF), and nasopharynx cells from severe COVID‐19 patients.^2^, ^4^, ^57^ T‐cell exhaustion‐associated genes were separated into “Activation,” “Function,” and “Regulation” categories (Figure 5C). *CD69*, *IFNG*, *TNF*, *IL2*, *TLR4*, *GZMB*, and *PRF1* were downregulated in severe COVID‐19 patients compared to moderate cases, suggesting loss of T‐cell activation, cytokine secretion, and cytotoxicity.^58^, ^59^ Moreover, increased expression of *IL10* may induce T‐cell dysfunction.^58^

Analyses of immune cell markers reflected decrease of T‐cell numbers in severe cases with downregulated *CD3*, *CD4*, and *CD8*, which was corroborated by large‐scale clinical studies^50^ (Figure 5D). We also observed a significant upregulation of *MZB1*, which agrees with a recent study demonstrating the unique role of plasmablasts in severe COVID‐19.

Because our functional enrichment analysis revealed an enrichment in miRNAs involved in TLR signaling, we set out to investigate the expression of *TLR* genes (Figure 5E). Most *TLR* mRNAs, including *TLR4*, *TLR5*, and *TLR8* were upregulated in the severe group, consistent with viral and bacterial co‐infection that was common among severe cases. Interestingly, two antiviral immunity‐associated *TLRs*, *TLR3* and *TLR7*, showed opposite expression profiles, which may account for diminished viral clearance and disease aggravation in severe cases.^60^

### 3.7 Global analysis of DEGs during COVID‐19 by bulk RNA‐seq

**FIGURE 6:**
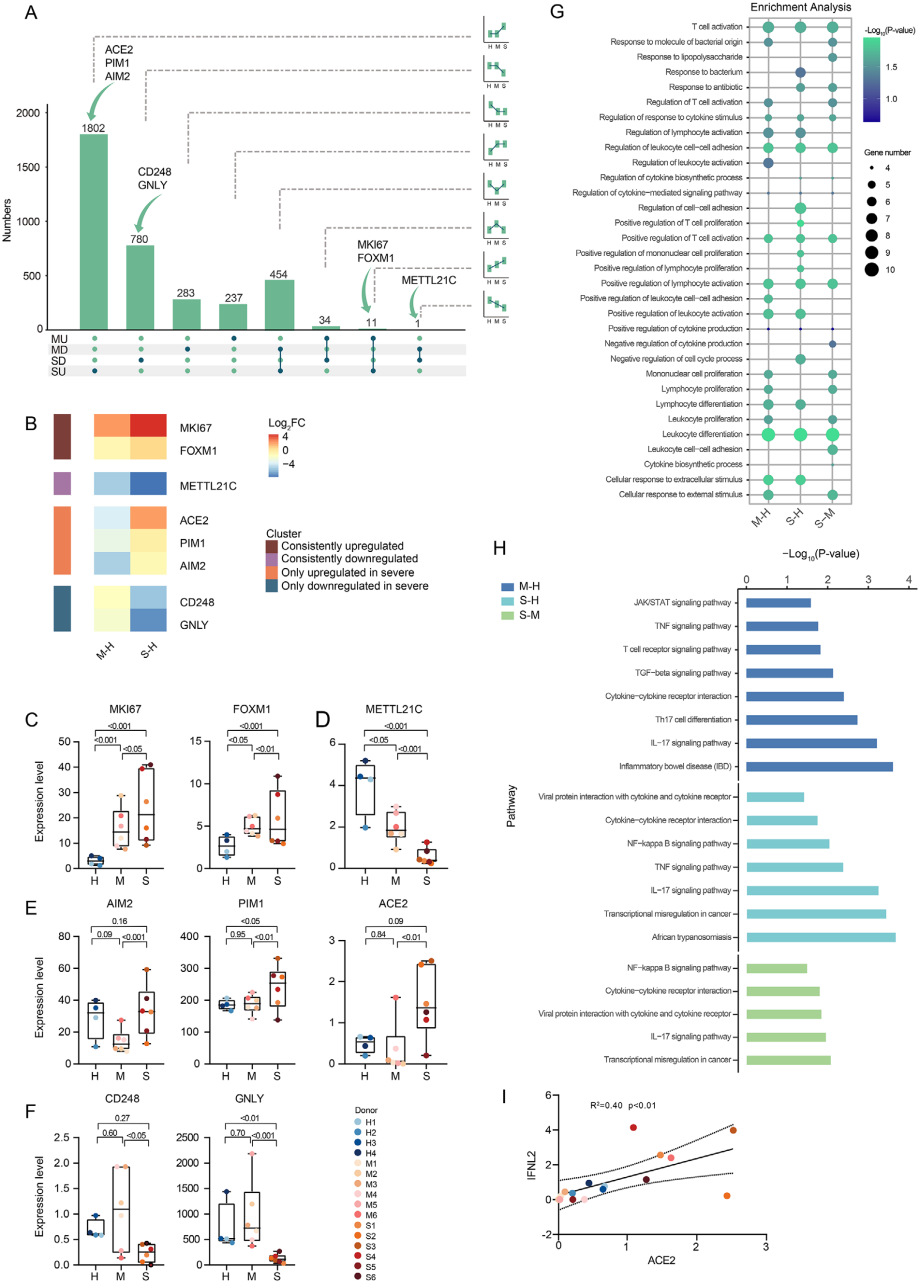
**Global analysis of DEGs identified by bulk RNA‐sEquation. (A)** UpSet plot shows the number of DEGs with eight expression tendencies in M‐H and S‐M comparisons. The bar and line charts show the tendency of each group. The arrows line out the position of the candidate mRNA. **(B)** The heatmap shows the candidate pathogenesis‐related mRNA. **(C‐F)** Expression of those mRNAs colored by donor of origin. Shown are *P*‐values generated by one‐way ANOVA with multiple comparisons by Tukey's test. The number of samples: H (n = 4), M (n = 6), and S (n = 6). Boxplot features: minimum whisker, the smallest value within; minimum box, 25th percentile; center, median; maximum box, 75th percentile; maximum whisker, the largest value within. **(C)** mRNAs consistently downregulated. **(D)** mRNAs consistently upregulated. **(E)** mRNAs only upregulated in severe COVID‐19 patients. **(F)** mRNAs only downregulated in severe COVID‐19 patients. **(G)** Dot plot depicts GO‐term functional enrichment by biological progress of three comparisons. **(H)** KEGG pathway enrichment of upregulated expressed genes of three comparisons. **(I)** Scatter plots shows the correlation between *ACE2* and *IFNL2*. The number of samples: M (n = 6) and S (n = 6)

Compared to miRNAs, the expression of mRNAs was more complex and characterized by less distinct DEG clusters (Figure S5B). Similar to our miRNA analysis, we searched for DEGs across the three comparisons (M‐H, S‐H, and S‐M) and obtained a list of DEGs possibly associated with disease pathogenesis (Figure 6A). Four groups of DEGs were identified (Figure 6B): (a) genes consistently upregulated in COVID‐19 patients, including the marker of cell proliferation, marker of proliferation Ki‐67 (*MKI67*),^61^ and regulator of macrophage function, forkhead box M 1 (*FOXM1*)^62^ (Figure 6C); (b) genes consistently downregulated, including methyltransferase like 21C (*METTL21C*) regulating NF‐κB signaling and the expression of *IL10* ^63^ (Figure 6D); (c) genes exclusively upregulated in severe COVID‐19 patients, including absent in melanoma 2 (*AIM2*), *PIM1*, and angiotensin I converting enzyme 2 (*ACE2*) (Figure 6E); cytoplasmic DNA can elicit *AIM2* expression to induce cell death^64^ while PIM1 kinase promotes airway inflammation;^65^ *ACE2* will be discussed in detail later; (d) genes exclusively downregulated in severe COVID‐19 patients, including CD8+ T‐cell inhibitor *CD248* ^66^ and cytotoxicity marker, granulysin (GNLY)^67^ (Figure 6F).

Functional enrichment analysis identified significant enrichments in the “response to bacterium” reflecting bacterial co‐infections and processes related to lymphocyte activation, proliferation, and regulation (Figure 6G). KEGG pathway analysis identified the “cytokine and cytokine receptor interaction” and “viral protein interaction with cytokine and cytokine receptor,” suggesting hypercytokinemia caused by severe infection (Figure 6H). Upregulation of “NF−κB signaling pathway” indicated the activation of common upstream pathways regulating cytokine production. The upregulated “IL‐17 signaling pathway” agreed with elevated levels of Th17 cells observed in COVID‐19 patients.^68^

Notably, *ACE2* was only upregulated in S‐M and S‐H comparisons (Figure 6E). *ACE2* encodes the angiotensin I converting enzyme 2, a cell receptor considered vital for the entry of SARS‐CoV‐2 in host cells.^69^ The scientific community has proposed that *ACE2* expression can be induced by interferon via STAT1 signaling^4^ and correspondingly, we observed the upregulation of *IFNL2* (Figure 6I). Interestingly, the fact that *ACE2* was mainly upregulated in epithelial cells coincided with the exclusive expression of interferon (IFN)‐λ receptor at the surface of epithelial cells.^70^ This observation may imply that upregulation of *ACE2* is induced by IFN‐λ and mediated by STAT1 signaling in lung tissues.

## 4 DISCUSSION

Here, we provide a comprehensive analysis of the noncoding and coding transcriptional landscape of the peripheral immune response in patients with COVID‐19. Our main findings were as follows: (a) miR‐146a‐5p, miR‐21‐5p, and miR‐142‐3p are potential biomarkers of COVID‐19 severity; (b) miR‐146a‐5p, miR‐21‐5p, and miR‐142‐3p are novel potential therapeutic targets for COVID‐19; (c) several miRNAs, such as miR‐15b‐5p, are specific for severe COVID‐19 and may serve as potential biomarkers and therapeutic targets; and (d) the blood transcriptome profiles suggest hyperactivation of the immune response, loss of T‐cell function, and immune dysregulation in patients with severe COVID‐19.

Researchers across the globe are pushing the boundaries of our understanding of COVID‐19 pathogenesis with multiple transcriptome sequencing technologies. Using bulk RNA‐seq of BALF samples, the immune signatures of COVID‐19 patients have been profiled, demonstrating robust innate immune responses with marked hypercytokinemia and increased IFN‐stimulated gene expression.^2^ Another recent study identified three different “immunotypes” associated with SARS‐CoV‐2 infection. It reported that patients with robust activation and proliferation of T cells and relatively exhausted CD8+ T‐cell responses seemed to have worse clinical outcomes.^71^ The host responses in the lower and upper respiratory tracts have also been studied using BALF and nasopharyngeal tissue, respectively. Characterization of BALF immune cells from patients with varying severity of COVID‐19 pointed toward the roles played by macrophages and CD8+ T cells in the disease.^72^ Research on nasopharyngeal tissue identified airway epithelial cell types and states associated with vulnerability to severe disease and demonstrated that macrophage‐epithelial cell interactions contribute to greater inflammation‐mediated tissue damage.^4^ The idea that critical diseases are associated with hyperinflammation and heightened immune effects has been systematically demonstrated. Recently, our team characterized the transcriptional changes occurring in PBMCs of COVID‐19 patients and demonstrated the presence of sustained hyperinflammation in recovered patients; our team also found that aging leads to immune system dysregulation and may partially account for COVID‐19 vulnerability in the elderly.^73^, ^74^

In addition to mRNAs, ncRNAs also play critical roles in several human diseases. To the best of our knowledge, in this study we describe for the first time an atlas of ncRNA expression in the RBC‐depleted whole blood of patients with moderate and severe COVID‐19.

Generally, miRNAs can influence the propagation of RNA viruses and disease pathogenesis in two ways—directly targeting the viral genome or regulating host immune response.^11^ One example of the first is miR‐122, which interacts with the genome of the hepatitis C virus, inhibiting viral RNA degradation in infected human liver cells. Using machine learning, researchers predicted the likelihood of miRNAs to target the SARS‐CoV‐2 genome. Interestingly, miR‐15b‐5p scored 99 in this assessment, suggesting high likelihood of direct binding.^25^ As to miRNAs impact on immune regulation, one example is the upregulation of miR‐146a in infections by EV17^75^ and dengue virus,^10^ which negatively regulates the host immune response. The influenza virus can also inhibit cytokine and chemokine responses in infected cells by inducing the production of miRNAs.^76^, ^77^ Here, we found that miR‐146a‐5p and miR‐21‐5p probably play opposite roles during SARS‐Cov‐2 infection. Their downregulation in COVID‐19 patients induces production of IRAK1, IRAK2, and TRAF6 and potentially elicits transcriptomic alterations leading to hyperactivation of the immune system and hyperinflammation.^15^, ^17^, ^43^, ^78^ In agreement with this hypothesis, the scRNA‐seq data indicates that downregulation of miR‐146a‐5p promotes *STAT1* expression, consistent with the heightened response to interferon signaling observed in nearly all cell types.^38^ miR‐21‐5p may also directly target *CCL20* and *MYC*, whose overexpression fosters the inflammatory response and the T‐cell metabolic reprogramming, respectively.^21^, ^40^, ^41^, ^79^ The relatively strong correlation between these two miRNAs and disease severity indicated that miR‐146a‐5p and miR‐21‐5p might be key contributors to COVID‐19 pathogenesis and serve as hub regulators of the host immune response. Interestingly, miR‐15b‐5p seemed to play a dual role. In addition to binding directly to SARS‐CoV‐2 genome, both datasets indicate that miR‐15b‐5p potentially induces T‐cell exhaustion by repressing the expression of *IFNG* and *CD69*.^23^, ^24^ These evidences suggest a key role for miR‐15b‐5p in COVID‐19 pathogenesis and patient deterioration. Researchers have demonstrated that previously mentioned miR‐122, directly targeting hepatitis C virus (HCV) genome, could also serve as an antiviral target for HCV infection treatment.^80^ Taking the potential roles miRNA biomarkers could play in COVID‐19 deterioration into consideration, these miRNAs might candidate biomarker miRNAs identified may as well serve as targets for COVID‐19 treatment.

Despite all the exciting potential clinical application of noncoding RNAs, several existing questions still require further address. First, several risk factors could influence the expression of peripheral ncRNAs, consequently confounding the accuracy of the biomarker ncRNAs. Previous research have indicated that almost all risk factors could potentially result in different expression of certain miRNAs in peripheral blood, including age,^81^ obesity,^82^ type 2 diabetes,^83^, ^84^ and coronary artery diseases^85^. Here, in this study, though we matched one of the most fundamental confounders, age, the impact of some other comorbidities was hard to control because of the complicated healthy condition background of each patients, especially those with severe COVID‐19. The diagnostic specificity of the noncoding RNA biomarkers is another consideration. Another problem is about the specificity of noncoding RNA biomarkers, since our study did not include non‐COVID‐19 patients with pneumonia or ARDS as positive controls for moderate and severe groups. Further researches including non‐COVID‐19 pneumonia and ARDS patients, as well as asymptomatic COVID‐19 patients were needed to validate the specificity of these biomarkers.

## 5 CONCLUSION

Overall, we provided a comprehensive atlas of the ncRNAs of peripheral immune cells in COVID‐19 patients. Our results revealed novel potential biomarkers and contributors to the pathogenesis and severity of COVID‐19. Several ncRNAs might participate in the hyperactivation of the immune response and inflammatory effects, loss of T‐cell function, and immune dysregulation in patients with severe COVID‐19. We believe that these findings will serve as a foundation for exploring in more depth the unknown facets of COVID‐19's etiology and a reference for the broad scientific community interested in expanding our understanding of this disease.

## CONFLICT OF INTEREST

The authors have declared no conflict of interest.

## ETHICS APPROVAL AND CONSENT TO PARTICIPATE

This study was approved by the Ethics Committee of the Huo Shen Shan Hospital of Wuhan.

## Supporting information

Figue S1

Figue S2

Figue S3

Figue S4

Figue S5

Figue S6

Table S1

Table S2

Table S3

## Data Availability

Sequencing data are available in the National Genomic Data Center (NGDC) (primary accession number HRA000238)
