# Supplementary figures and images for: The noncoding and coding transcriptional landscape of the peripheral immune response in patients with COVID‐19
Source: Clin Transl Med. 2020 Oct 11;10(6):e200. doi: 10.1002/ctm2.200 (PMC7548099; doi:10.1002/ctm2.200)

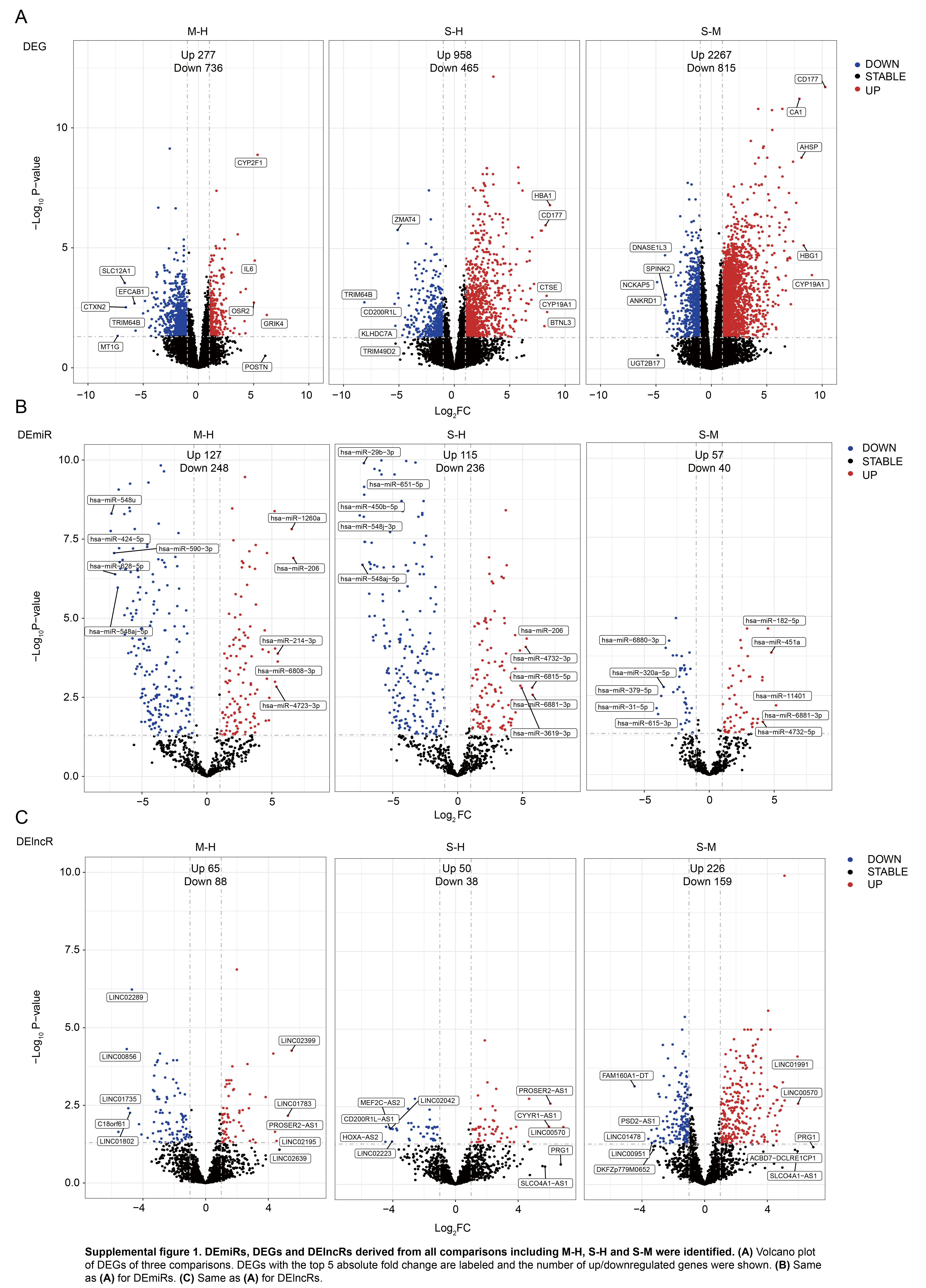

Supplement: Supplementary file 1 — Figue S1 [file CTM2-10-e200-s001.tif]

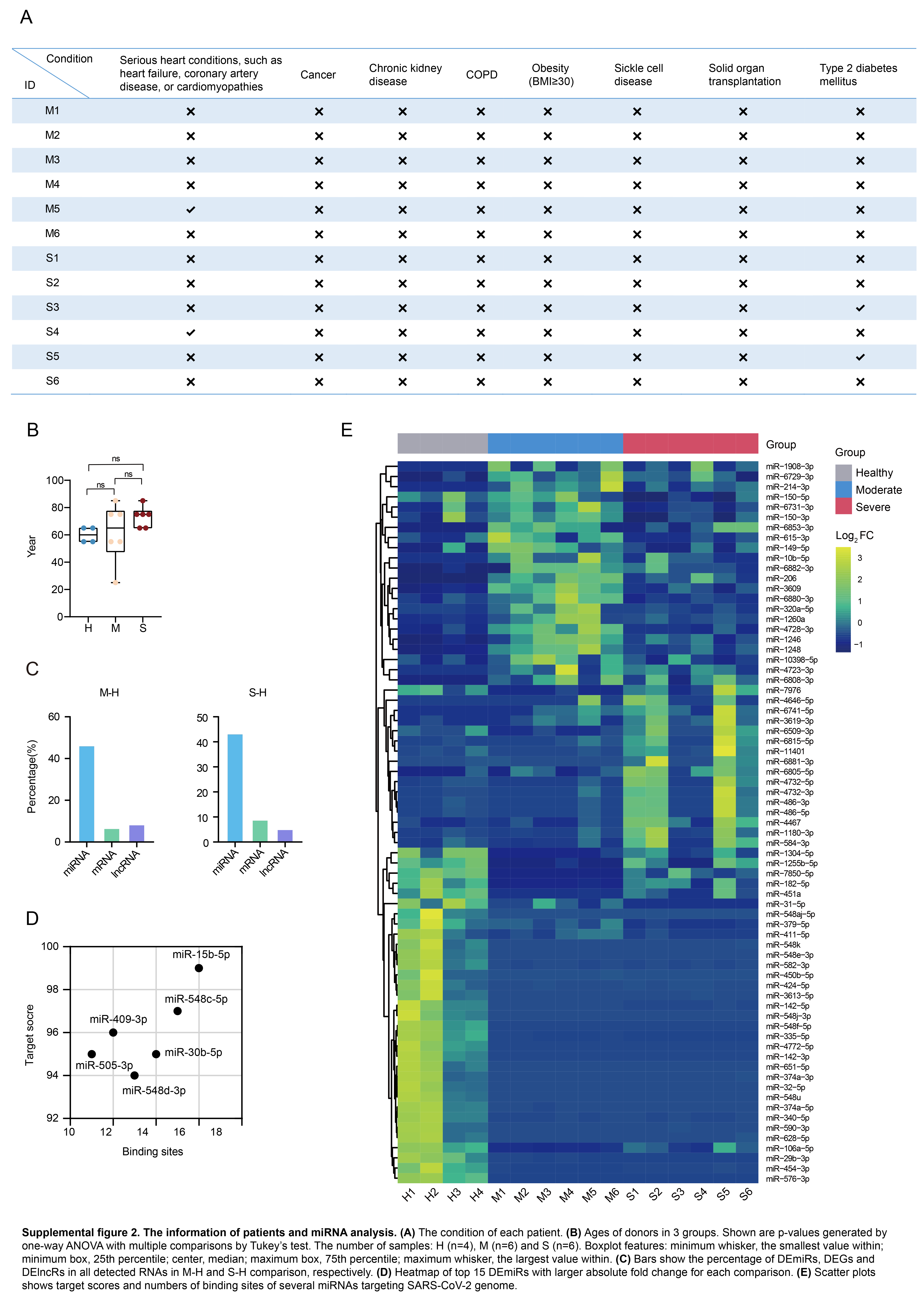

Supplement: Supplementary file 2 — Figue S2 [file CTM2-10-e200-s002.tif]

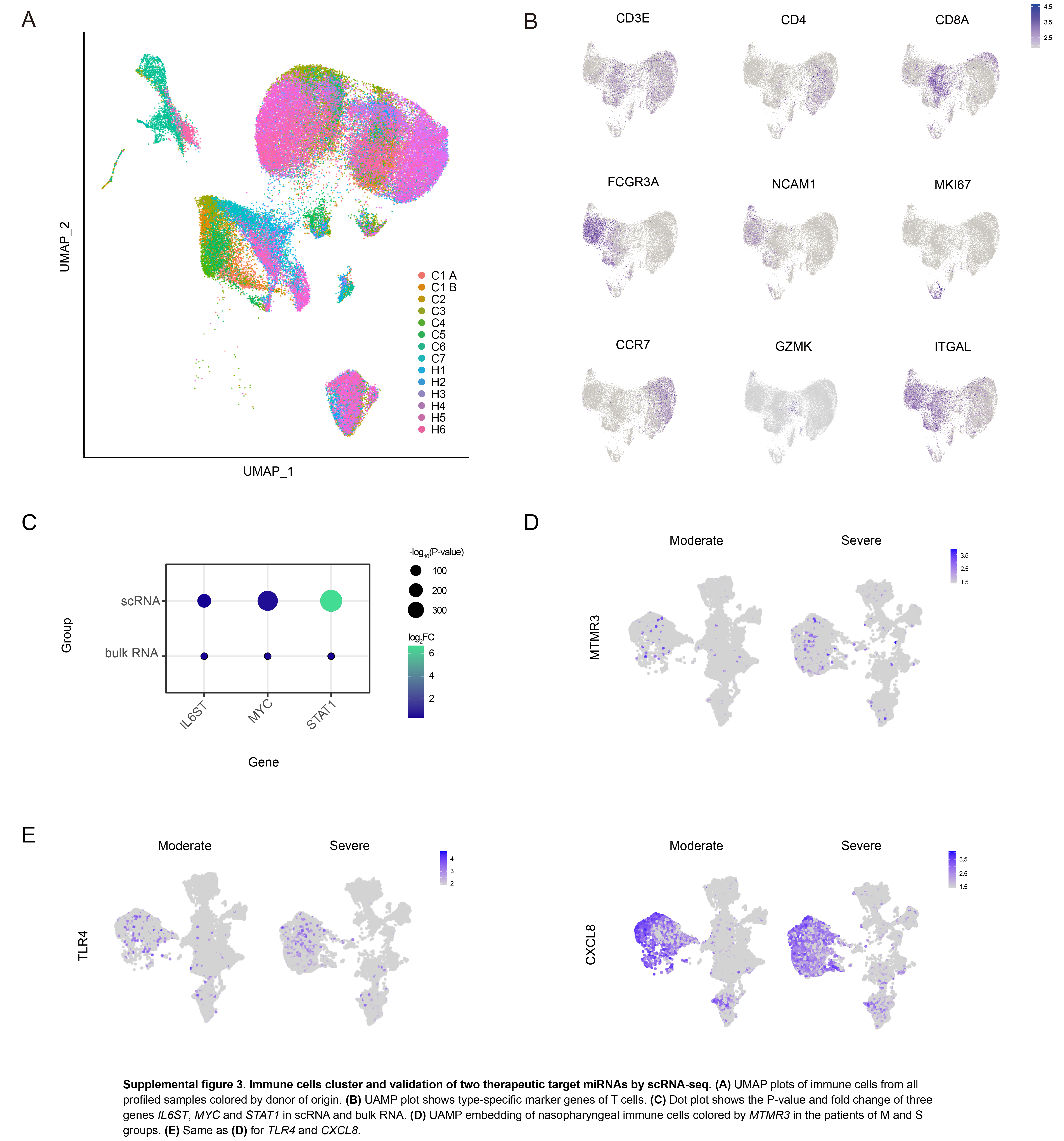

Supplement: Supplementary file 3 — Figue S3 [file CTM2-10-e200-s003.tif]

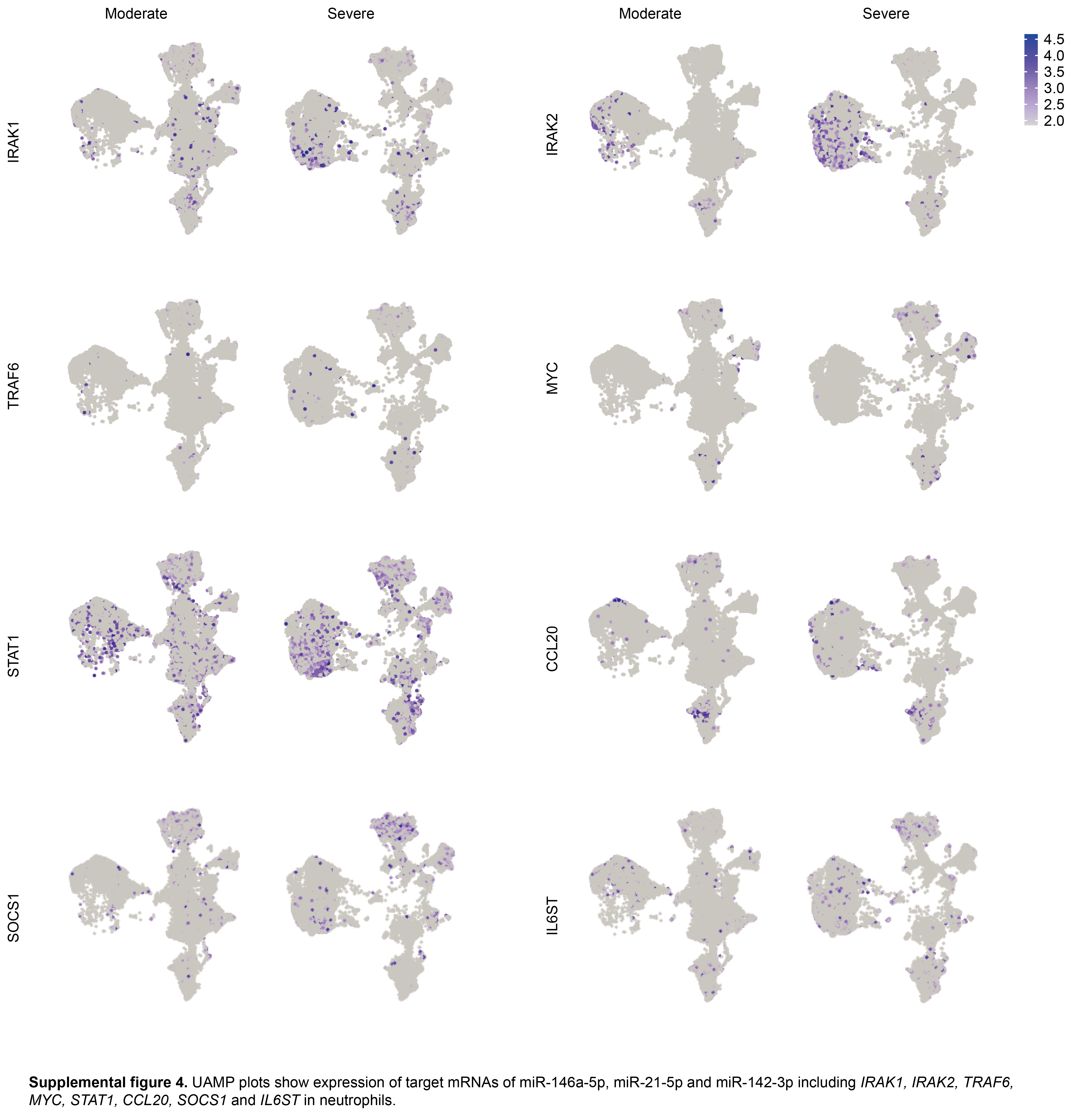

Supplement: Supplementary file 4 — Figue S4 [file CTM2-10-e200-s004.tif]

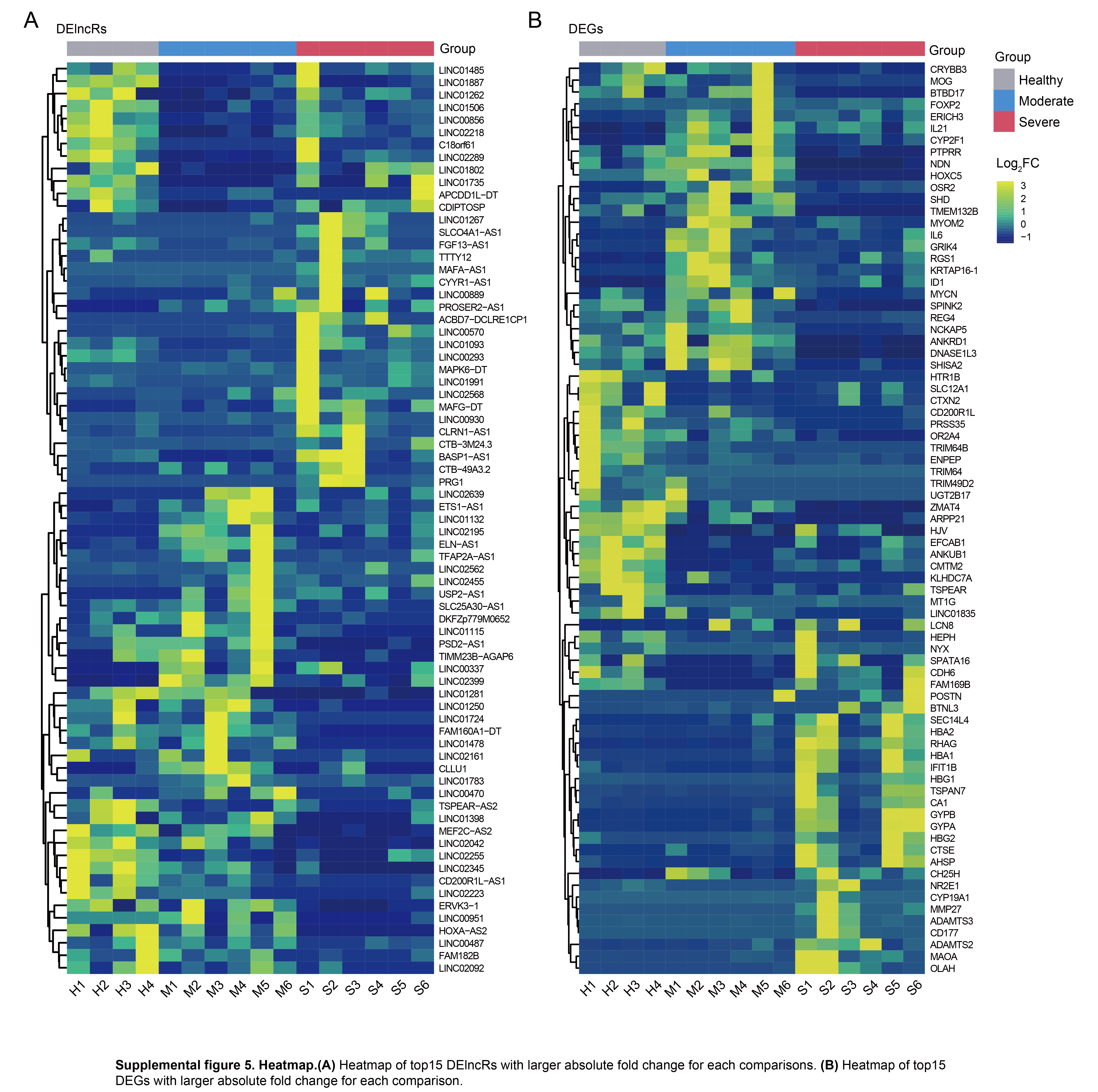

Supplement: Supplementary file 5 — Figue S5 [file CTM2-10-e200-s005.tif]

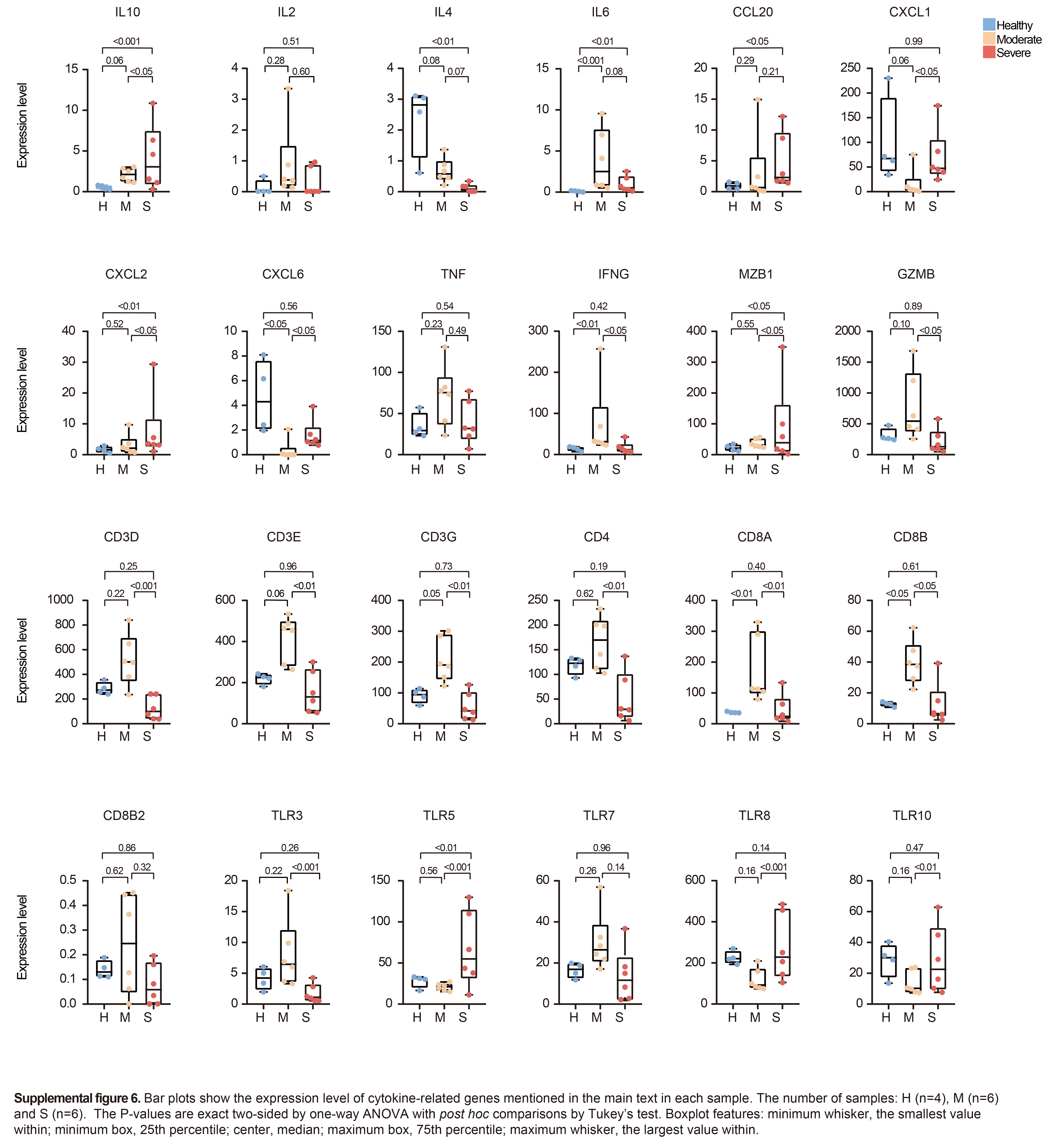

Supplement: Supplementary file 6 — Figue S6 [file CTM2-10-e200-s006.tif]
